# Steering surface topographies of electrospun fibers: understanding the mechanisms

**DOI:** 10.1038/s41598-017-00181-0

**Published:** 2017-03-13

**Authors:** Gökçe Yazgan, Ruslan I. Dmitriev, Vasundhara Tyagi, James Jenkins, Gelu-Marius Rotaru, Markus Rottmar, René M. Rossi, Claudio Toncelli, Dmitri B. Papkovsky, Katharina Maniura-Weber, Giuseppino Fortunato

**Affiliations:** 1 Empa, Swiss Federal Laboratories for Materials Science and Technology, Laboratory for Protection and Physiology, CH-9014 St. Gallen, Switzerland; 2 Empa, Swiss Federal Laboratories for Materials Science and Technology, Laboratory for Biointerfaces, CH-9014 St. Gallen, Switzerland; 3 School of Biochemistry and Cell Biology, University College Cork, College Road, Cork, Ireland; 4 Empa, Swiss Federal Laboratories for Materials Science and Technology, Center for X-ray Analytics, CH-8600 Dübendorf, Switzerland

## Abstract

A profound understanding of how to tailor surface topographies of electrospun fibers is of great importance for surface sensitive applications including optical sensing, catalysis, drug delivery and tissue engineering. Hereby, a novel approach to comprehend the driving forces for fiber surface topography formation is introduced through inclusion of the dynamic solvent-polymer interaction during fiber formation. Thus, the interplay between polymer solubility as well as computed fiber jet surface temperature changes in function of time during solvent evaporation and the resultant phase separation behavior are studied. The correlation of experimental and theoretical results shows that the temperature difference between the polymer solution jet surface temperature and the dew point of the controlled electrospinning environment are the main influencing factors with respect to water condensation and thus phase separation leading to the final fiber surface topography. As polymer matrices with enhanced surface area are particularly appealing for sensing applications, we further functionalized our nanoporous fibrous membranes with a phosphorescent oxygen-sensitive dye. The hybrid membranes possess high brightness, stability in aqueous medium, linear response to oxygen and hence represent a promising scaffold for cell growth, contactless monitoring of oxygen and live fluorescence imaging in 3-D cell models.

## Introduction

The high surface to volume ratio of electrospun fibers and hence their high specific surface area allows their use in a great variety of applications. A further increase of the surface area by incorporating tailored surface topographies is therefore of major interest for many industrial and biomedical fields of applications including tissue engineering, drug delivery, catalysis, filtration and sensors^1–4^. To alter the surface topography of e-spun fibers, two different principles have emerged: (1) post-processing procedures to add structural components or to selectively remove compounds from the fibers, (2) exploitation of phase separation processes during fiber formation aiming at selective and time dependent drying and crystallization processes. The first approach, controlled polymer crystallization on electrospun fibers either by incubation in polymer solution or by polymer solvent evaporation, creates lamellae on fibers with e.g. shish-kebab structures on fiber surfaces^5–7^. Alternatively, solvent extraction^8–10^, salt leaching^11^ or calcination of electrospun fibers made up of two components yields porous surfaces by selective removal of the sacrificial component^12^. The second approach, structuring by *in-situ* spinning procedures, has been demonstrated by electrospinning in a humid environment^13–20^, into liquid nitrogen^21^ or into non-solvent baths^22, 23^ to result in porous fiber structures. Various attempts, mainly being inspired from porous polymer film formation techniques, have been made to explore underlying principles generating such surface structures of electrospun fibers. For instance, Srinivasarao *et al*.^24^ explain the ordered and monodispersed sized pores formed on polymer casted films to originate from breath figures. These represent imprints left by water droplets that condense on casted films due to evaporative cooling and have also been proposed to induce same characteristics on electrospun fibers^18^. On the other hand, the temperature drop caused by evaporative cooling of polymer solutions can result in thermally induced phase separation described as one of the porous membrane preparation techniques^25^. It is also argued as a possible reason behind the polymer jet in electrospinning separated into polymer rich and polymer lean regions^13, 18, 26^. Upon complete drying of the fibers, polymer lean regions are then expected to lead to the formation of inner pores or surface topographies. Further, thermodynamic instability can be introduced either by water from the humid environment acting as a non-solvent or by the addition of a non-solvent directly into the polymer solution mixture^17, 26–29^. Thus, to create fibers with desired surface topographies, it is of crucial importance to properly choose a specific solvent and solvent mixture for each polymer. However, the most commonly used approach is to empirically select the solvents with defined volatility, water miscibility, dielectric constant and conductivity by trial and error. A more efficient approach is to thermodynamically predict the initial degree of solubility of a polymer in different solvents or solvent mixtures by use of solubility parameters^30–32^.

Among the different solubility models^33^, Hansen Solubility Parameters are widely used to predict material properties such as the affinity of substances to each other, gelation behavior of organogels^34^, encapsulation efficiency of drugs^35^, barrier function of release systems^36^ as well as good and poor solvents for polymers^30, 37^.

Polycaprolactone (PCL), a biodegradable, FDA approved, semi-crystalline polyester which has been shown to be biocompatible for specific applications in the areas of drug delivery, medical sutures, dentistry, wound dressings as well as tissue engineering^38^, has been extensively studied in the electrospinning field. Although it has been explored for solution, blend and emulsion electrospinning, *in-situ* structuring of PCL fibers has only recently been under investigation^19, 20, 27, 28^. However, elaborated investigations are required to advance the understanding of the pore formation mechanism during fiber formation.

Here, we demonstrate how electrospun fiber surface structure formation correlate with theoretically predicted polymer solubility alterations in different solvents during their evaporation, the corresponding solvent evaporation rates, subsequent polymer jet surface temperature changes and thus the inevitable influence on the solution thermodynamic equilibrium. As a model system PCL was used and spun at selected environmental humidity conditions. The extension of our findings to other polymer-solvent systems can establish a universal prediction tool for surface morphology evolution of electrospun fibers as shown for another hydrophobic polymer, poly L-lactic acid (PLLA) as well as for a hydrophilic polymer, polyvinylpyrrolidone (PVP).

To highlight the application potential of developed porous morphologies in areas such as tissue engineering and biosensing, we have investigated the oxygen sensing ability of nanoporous structures in which a phosphorescent dye, Pt(II)-tetrakis (pentafluorophenyl) porphine (PtTFPP), was embedded in the PCL matrix after fabrication of the fibres^39^. This composite was tested as 3-D scaffold for localized oxygen monitoring in culture of cancer cells.

## Experimental Section

### Materials

Polycaprolactone (PCL) (70,000–90,000 g mol^−1^), Polyvinylpyrrolidone (PVP) (360,000 g mol^−1^), dichloromethane (DCM) and dimethyl sulfoxide (DMSO) were obtained from Sigma–Aldrich (Switzerland), Polylactic acid (PLLA) (Ingeo™ Biopolymer 2500HP) from Natureworks, N,N- Dimethylformamide (DMF) from VWR Chemicals, Ethanol (without additive, ≥99.8%) and o-xylene from Fluka Analytical, chloroform (CHCl_3_, ≥99%) from Fischer Scientific and methanol (CH_3_OH, ≥99%) from Fluka. PtTFPP was from Frontier Scientific (USA). Calcein Green AM, cell growth medium, acetone, Na_2_SO_3_, KH_2_PO_4_, phosphate-buffered saline (PBS) and all the other reagents were from Sigma-Aldrich (Dun Laoghaire, Ireland). All materials were used without any further purification.

### Polymer solutions preparation and spinning procedures

PCL solutions were prepared at a concentration of 15% (w/v) by using pure solvents DCM, CHCl_3_, and solvent mixtures of CHCl_3_:MeOH with a volume ratio of 18:2 and CHCl_3_:DMSO with volume ratios of 19:1, 18:2 and 16:4, respectively. After stirring overnight, the surface tension of each solution was determined in triplicates by the pendant drop method using optical contact meter equipment (Krüss GmbH, Germany). Additionally, electrical conductivity of the solutions was measured using a conductometer (Metrohm 660, Switzerland). PLLA solutions were prepared at a concentration of 10% (w/v) by using solvent mixtures of CHCl_3_:MeOH with volume ratios of 18:2 and 17:3 and CHCl_3_:DMF with volume ratios of 17:3, respectively. PVP solutions were prepared at a concentration of 10% (w/v) by using EtOH and a solvent mixture of EtOH:Xylene with the volume ratio of 14:6. PCL nanofibers were prepared according to the study of Guex *et al*.

All solutions were processed into fibers at three different relative humidity (RH) conditions, 12, 35 and 55%, respectively, by a custom-built electrospinning set-up as described previously^40^. Flow rate was held at 30 µL/min for all solutions and a potential of +8 kV on the needle and −3 kV on the counter electrode for PCL solutions with a fixed tip-to-collector distance of 25 cm was applied. The electrospinning process parameters were set as +12, −3 KV with 20 cm of distance for PLLA and as +7, −4 with 15 cm of distance for PVP solutions.

### Characterization of fibers

Fiber diameters and their distribution as well as the inner and surface morphology of the electrospun fibers were investigated by Scanning Electron Microscopy (SEM) (Hitachi S-4800, Hitachi High-Technologies, USA & Canada) using 2 kV accelerating voltage and 10 mA current flow. To examine the surface morphology, the sample patches were mounted on metal stubs with double-sided adhesive tape. In order to image the inner fiber morphology, cross sections were obtained by cutting fibers in liquid N_2_ with a surgical blade and the cut pieces were placed on a sample holder with a vertical sample fixation unit. Prior to loading, the samples were sputter coated with gold/palladium of 5 nm thickness to increase the electrical conductivity. The mean fiber diameters were calculated based on measurements of 90–100 fibers from the SEM micrographs using Image J software.

Wide angle X-ray scattering (WAXS) experiments were performed on a STOE Imaging Plate Diffraction System IPDS II, using Mo Kα (λ = 0.71 Å) radiation in transmission geometry. The sample-to-detector distance was set to 200 mm allowing collecting data up to 2Θ = 38°. The diffraction patterns were acquired by means of an image plate detector system with exposure times of 15 min at room temperature. In order to calculate the crystallinity, the two dimensional scattering patterns were radially integrated into one-dimensional scattering functions, background subtracted, followed by a peak fitting to Pearson7 functions using Fityk 0.9.8 software^41^.

### Hansen Solubility Parameters

PCL solubility in various solvents and solvent mixtures, relative solvent evaporation rates from these solutions, the consequent change in the Relative Energy Distance (RED) of solvent mixtures and the approximate solvent temperature change due to evaporation were calculated by use of the software Hansen Solubility Parameters in Practice (HSPiP) which has been developed by Steven Abbott, Charles M. Hansen and Hiroshi Yamamoto^42^.

As an approximation, the model which is introduced within this software accounts for the published Relative Evaporation Rates (RER) of the solvents, which are determined in comparison to the evaporation of standard solvent Butyl acetate (assuming that it evaporates in 100 time units) together with activity coefficients to simulate the evaporation rate of the solvents^42^. The RER calculations are implemented to electrospinning and fiber morphology correlation with the following assumptions: i) diffusive evaporation^43^ of the solvent, which is the main mechanism of solvent evaporation, takes place mainly from the straight region of the initiated jet from the tip and ii) the forced convective mass transfer of the solvent that is a result of the whipping motion is neglected to simplify the complex electrospinning process.

The evaporation of solvents from a polymer solution prepared with a solvent mixture can alter the RED depending on the volatility differences of the individual solvents. These changes in RED values were simulated during the evaporation in function of time and the corresponding plots were generated.

Additionally, solvent evaporation as an endothermic process removes heat from the solution and therefore causes a temperature decrease on the jet surface. The computed reduced fiber jet surface temperature is defined as the wet bulb temperature (*T*_*wb*_) and calculated iteratively (Supplementary Fig. S1) by use of the energy balance as equation (1) at selected standardized time points^42^:

1 $${C}_{a}\times ({T}_{a}-{T}_{wb})={\rm{\Delta }}{H}_{vap}\times {\rm{\Delta }}M$$

where *C*_*a*_ is the heat capacity of air at a given environmental temperature, *T*_*a*_ and *T*_*wb*_ are the temperature values of the air and the solvent, respectively, ΔH_*vap*_ is the latent heat of vaporization of the solvent and Δ*M* is the evaporated amount of solvent. In this way, a simulation of the evaporation of solvent and solvent mixtures as well as the consequent changes of the RED values and jet surface temperatures in function of a standardized time were calculated to understand the fiber formation process with respect to surface structuring.

### Evaluation of PCL membranes for O_2_ sensing

For dye impregnation, three types of PCL membranes, which were made of microfibers either with porous or smooth surface morphologies and nanofibers with smooth surface morphology (Supplementary Fig. S6a), were incubated at room temperature in acetone:water (7:3) mixture containing 0.025 mg/mL of PtTFPP (1 h), dried overnight and washed sequentially with mixtures of acetone:water (3.5:6.5), (1.5:8.5) and deionized water. Membranes were stored in the dark at room temperature in dried form and were pre-soaked in 70% ethanol followed by equilibration in phosphate-buffered saline, pH 7.4 (PBS) or cell growth medium before the experiments. For analysis of stability, stained membranes were incubated either in PBS or cell growth medium (10% FBS, McCoy 5 A, 10 mM HEPES-Na, pH 7.2) under sterile conditions (37 °C) over 7 days period, then PBS and cell growth medium were transferred on 96-well flat bottom microplate and measured on time-resolved fluorescence microplate reader Victor2 (Perkin Elmer) using D340 excitation and 642 nm emission filters (delay time 30 μs).

Human colon carcinoma HCT116 cells (ATCC) were handled as described previously^44^: briefly, cells were seeded on ethanol pre-soaked membranes in cell growth medium at concentration 0.5 * 10^6^ cells per 35 mm Petri dish containing membrane. After 48 h of cultivation, cells were counter-stained with Calcein Green (1 µM, 30 min), washed once and proceeded to live cell imaging. Confocal phosphorescence lifetime imaging microscope (PLIM) was performed on an upright fluorescence microscope Axio Examiner Z1 (Carl Zeiss) with 20x/1.0 W-Plan Apochromat and Fluar 5x/0.25 objectives, heated incubator (37 °C), motorized Z-axis control, DCS-120 confocal TCSPC scanner (Becker & Hickl GmbH), R10467U-49 photon counting detector (Hamamatsu photonics) and dedicated SPCI software (Becker & Hickl GmbH) essentially as described previously^44^. The O_2_ calibration was performed using 390 nm LED excitation (650 nm emission) on inverted widefield PLIM microscope (Zeiss-LaVision), equipped with integrated CO_2_/O_2_/T control (Pecon)^45^. To achieve complete deoxygenation in PBS, a solution of 5 mg/ml KH_2_PO_4_; 5 mg/ml Na_2_SO_3_ was added to the membranes. Experiments were performed in triplicate.

### Statistical Analysis

All data were expressed as mean ± standard deviation of at least three samples. One-way analysis of variance (ANOVA) was performed to determine statistical significance of the data using Origin 9.1. A value of *p* < 0.05 was considered statistically significant.

## Results

### Polymer solubility determined by HSP

**Figure 1:**
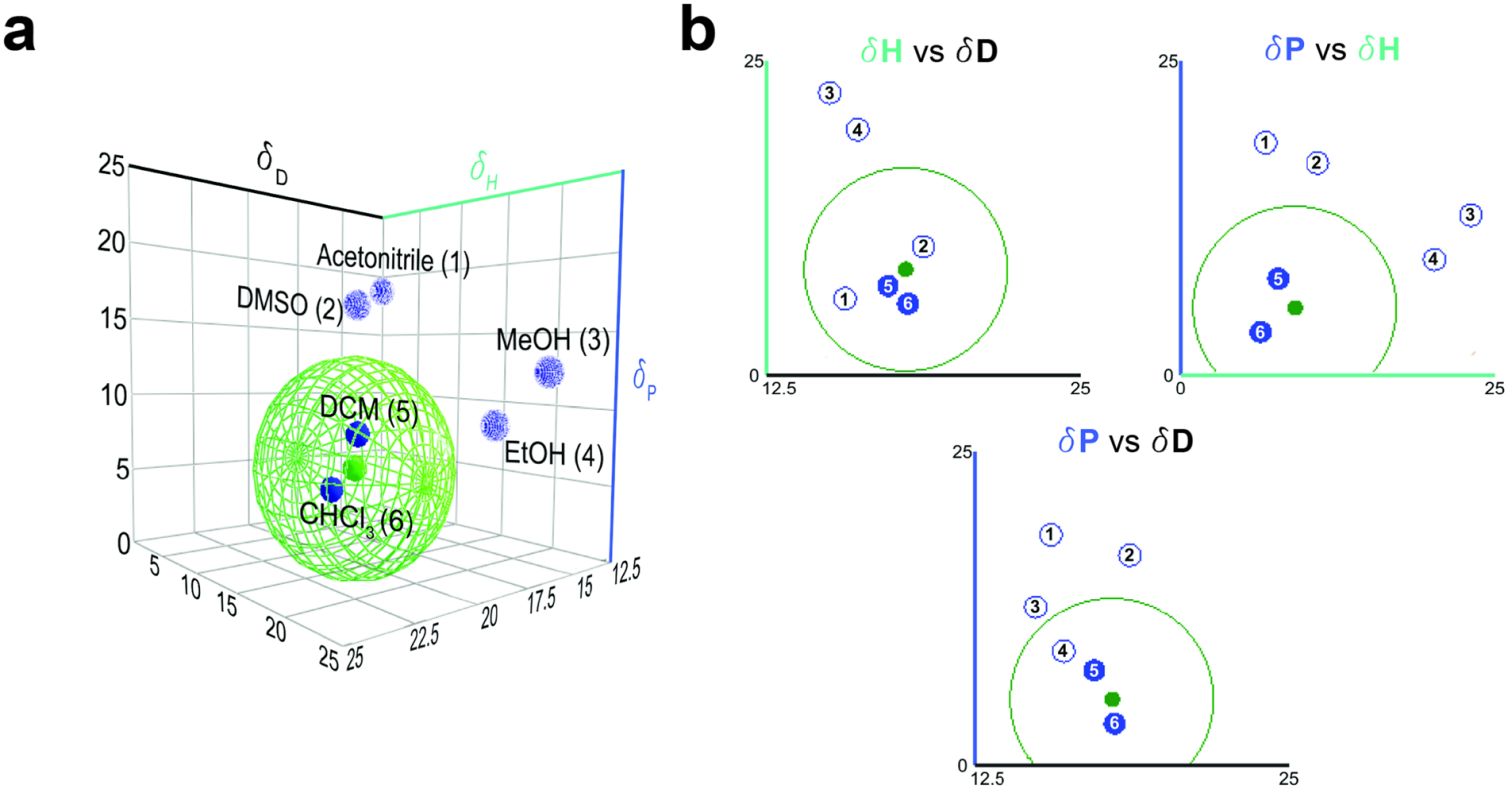
Polymer and solvents in the 3-D Hansen space: (**a**) PCL solubility sphere and positions of solvents visualized in 3-D Hansen space; non-solvents Acetonitrile (1), DMSO (2), MeOH (3), EtOH (4) are located outside of the PCL sphere, good solvents DCM (5) and CHCl3 (6) inside the PCL sphere (**b**) solvents (Acetonitrile (1), DMSO (2), MeOH (3), EtOH (4), DCM (5) and CHCl_3_ (6)) located in 2D projected planes.

The HSP theory, which has been developed by Hansen, derives its origin from the cohesive energy density (E/V) of a liquid that is the energy of vaporization per volume unit^46^. The solubility parameter, δ, is defined as the square root of this cohesive energy, which reflects the degree of attractive forces holding the molecules together. Hansen subdivided this total solubility parameter into three fractions: dispersive interactions (δ_D_), polar interactions (δ_P_) and hydrogen bonding (δ_H_) in units of MPa^½^. These three parameters can be visualized as coordinates in a 3-dimensional diagram termed 3-D Hansen space (Fig. 1) in the HSPiP^42^ software. It allows a good illustration of the compatibility extent of different materials by evaluating their distance to each other. The distance (R_a_) is formulated as equation (2):

2 $${R}_{a}=\sqrt{4{({\rm{\Delta }}{\delta }_{D})}^{2}+{({\rm{\Delta }}{\delta }_{P})}^{2}+{({\rm{\Delta }}{\delta }_{H})}^{2}}$$

In 3-D Hansen space, solvents are depicted as single points whereas the polymers are represented as spheres in which solvents with good solving or swelling properties for that polymer are located. The coordinate position of a substance with respect to a polymer solubility sphere is characterized by the ratio of the coordinates’ distance to the center of the sphere and the sphere’s interaction radius^35^. This ratio is called the RED^46^. A RED value lower than 1.0 indicates a high affinity or solubility (coordinate position within the sphere) whereas a RED value higher than 1.0, lower affinity to the polymer (coordinate position outside the sphere).

We constructed and visualized the solubility sphere of our model polymer, PCL, with its center coordinate values (δ_D_ = 17.7 MPa^½^, δ_P_ = 5.0 MPa^½^ and δ_H_ = 8.4 MPa^½^) and interaction radius (R = 8) value taken from the HSPiP software^42^. In order to verify the HSP theory, experimentally known good solvents and non-solvents of PCL were selected^32, 47^ and the RED values of these common electrospinning solvents such as CHCl_3_, DCM, DMSO, ethanol (EtOH), acetonitrile and methanol (MeOH) were calculated. Values for CHCl_3_ and DCM are located inside this sphere with RED values of 0.37 and 0.41, respectively. Values for DMSO, EtOH, Acetonitrile and MeOH were located outside of the PCL sphere due to RED values of 1.45, 1.53, 1.76 and 2.10, respectively (Fig. 1).

Based on these results, both good solvents of PCL, DCM and CHCl_3_, were chosen to study the effect of solvent evaporation rate on the final fiber morphology at three different RH conditions. Furthermore, the two non-solvents DMSO and MeOH, which have different evaporation rates in comparison to CHCl_3_, were chosen to mix with CHCl_3_ in order to investigate their influence on the final fiber morphology at three different RH conditions.

### Influence of solvent evaporation rate and RH on final fiber morphology

**Figure 2:**
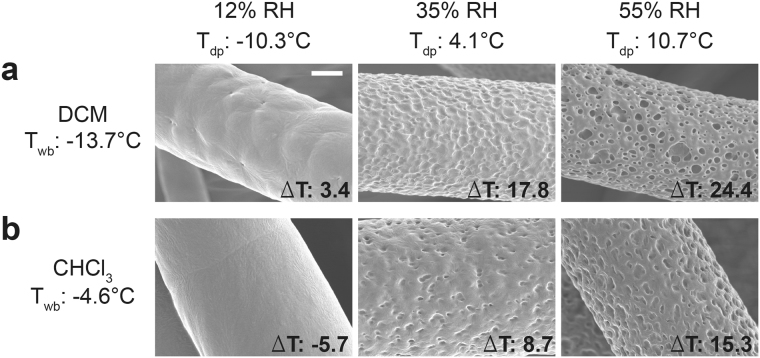
Morphologies of electrospun PCL fibers spun with DCM and CHCl_3_: SEM micrographs and corresponding Δ*T*(*T*_*dp*_ − *T*_*wb*_) of (**a**) DCM (upper row) and (**b**) CHCl_3_ (lower row) solution spun PCL fibers at 12%, 35% and 55% RH, respectively (scale bar: 2 µm).

In order to study the relation between solvents with different evaporation rates and final fiber morphologies, solutions with the PCL solvents DCM and CHCl_3_ were prepared. Both solutions were electrospun in a controlled environment at 20 °C and three different RH conditions of 12%, 35% and 55%, respectively. Fibers electrospun from DCM solution yielded slightly structured surfaces when processed at 12% RH, shallow pits covered surfaces of fibers that were produced at 35% RH and more distinct and deeper structures were obtained on fibers spun at an RH of 55% (Fig. 2a) with average fiber diameters of 7.0 ± 1.3 µm, 7.0 ± 1.0 µm and 7.4 ± 1.0 µm, respectively. Electrospinning of the PCL solution prepared with CHCl_3_ at 12% RH resulted in fibers with completely smooth surfaces (Fig. 2b). Distinctly separated structures on the fiber surface were formed when it was electrospun at 35% RH and more pronounced structural units occurred at 55% RH (Fig. 2b) with average fiber diameters of 8.7 ± 0.9 µm, 8.2 ± 0.8 µm and 8. 8 ± 0.7 µm, respectively.

Electrospinning is a complex, multiphysics process involving electrohydrodynamics, mass and heat diffusion and transfer, and solidification^43^. Generation of fibers in electrospinning is a result of solvent evaporation from the jet surface both in the straight regime and in the whipping instability regime. In our simulations for solvent evaporation from the polymer solution, the complex electrospinning process was simplified by use of diffusive evaporation scenarios and the relative evaporation rates of solvents to the reference solvent, butyl acetate, was used. DCM evaporated faster and within 15 time units (a.u.) whereas CHCl_3_ evaporated with a slower rate and within 25 time units (a.u.).

When solvent evaporation takes place, the temperature of the polymer solution surface decreases due to the latent heat loss to a minimum temperature defined as the wet-bulb temperature (*T*_*wb*_)^42^. Besides, the temperature to which air can be cooled down reaching full saturation is the dew point (*T*_*dp*_). Based on solution evaporation simulations, *T*_*wb*_ can decrease to a theoretical minimum of −13.7 °C in the case of DCM but only to −4.6 °C in the case of CHCl_3_ (Fig. 2). Moreover, the *T*_*dp*_ of the air at 12% RH is −10.3 °C while it is 4.1 °C and 10.7 °C at 35% and 55% RH, respectively (Fig. 2).

When saturated air encounters a surface colder than its temperature, water condensation is expected. Hence, water condensation condition at the air polymer interface can be formulated as Δ*T* = (*T*_*dp*_ − *T*_*wb*_) > 0. Accordingly, Δ*T* of the DCM solution increased from 3.4 °C at 12% RH to 17.8 °C at 35% RH and to 24.4 °C at 55% RH (Fig. 2a). Besides, Δ*T* of CHCl_3_ solution at 12% RH was below zero (−5.7 °C) and it increased to 8.7 °C and 15.3 °C at 35% and 55% RH, respectively (Fig. 2b).

The fiber surface structure changed from smooth to textured topography in the case of CHCl_3_ for which Δ*T*(*T*_*dp*_ − *T*_*wb*_) rose above zero and the structures evolved with the increase in Δ*T*. The correlation between the calculated temperature difference and the resulting fiber surface structure formation is shown for the first time to the best of our knowledge. Additionally, Δ*T* values of DCM solutions were above zero at all RH conditions and this was also reflected as progressing structured fiber surfaces with increasing Δ*T* values following a RH increase.

### Influence of non-solvent evaporation rate and RH on final fiber morphology

**Figure 3:**
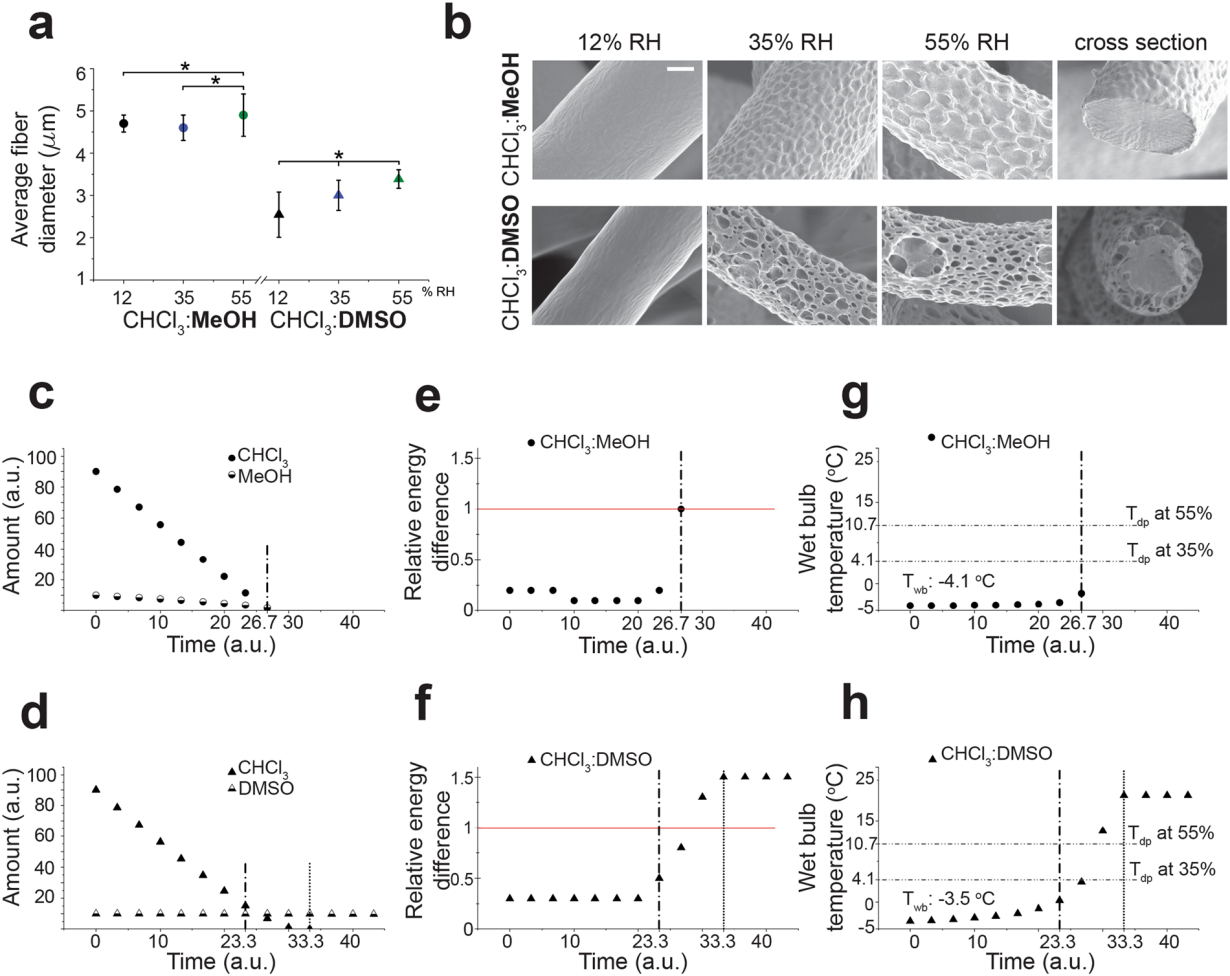
Resulting PCL fiber morphologies, simulated solvent evaporation and RED value changes during fiber formation for the solvent systems CHCl_3_:MeOH and CHCl_3_:DMSO: (**a**) Comparison of average fiber diameter of electrospun fibers from CHCl_3_:MeOH and CHCl_3_:DMSO solutions. Data represent means ± s.d. *P < 0.05; ANOVA One-way test, (**b**) SEM micrographs of electrospun fibers from CHCl_3_:MeOH (upper row) and CHCl_3_:DMSO (lower row) solutions at 12, 35, 55% RH, respectively (scale bar: 1 µm), (**c**,**d**) simulated evaporation of CHCl_3_ and MeOH and CHCl_3_ and DMSO, respectively from PCL solutions (assuming the initial total amount of a solution is 100 parts and the solvent amount changes by evaporation during electrospinning), (**e**,**f**) simulated RED value change during solvent evaporation from CHCl_3_:MeOH and CHCl_3_:DMSO solutions, respectively (**g**,**h**) wet bulb temperature change (°C) during solvent evaporation from CHCl_3_:MeOH and CHCl_3_:DMSO solutions, respectively, (dash-dot line) last time point of similar amounts of solvent (S) and non-solvent; (round dot line) time point S = 0; (red line) solubility limit for the polymer in the solvent system).

To explore the relation between the addition of non-solvent into the polymer solution and the subsequent changes on the electrospun fibers, polymer solutions prepared with solvent mixtures of CHCl_3_:MeOH (18:2) and CHCl_3_:DMSO (18:2), which have the same ratio of solvent to water miscible non-solvents, were compared. The initial RED values of the solvent mixtures with respect to PCL were calculated as 0.2 and 0.3 for CHCl_3_:MeOH and CHCl_3_:DMSO, respectively by use of the weighted volume average of individual solvents. Fibers spun from CHCl_3_:MeOH (18:2) mixture had 4.7 ± 0.2 µm, 4.6 ± 0.3 µm and 4.9 ± 0.5 µm of average fiber diameter at increasing RH conditions. On the other hand, fibers spun from CHCl_3_:DMSO (18:2) mixture had lower average fiber diameters of 2.5 ± 0.5 µm, 3.0 ± 0.4 µm and 3.4 ± 0.2 µm at increasing RH conditions (Fig. 3a). Conductivity, surface tension and viscosity of both solutions were measured as these contribute to electrospinnability of a solution as well as the final fiber diameter. The difference between solutions for none of the properties was statistically significant to correlate with the fiber diameter changes (Supplementary Fig. S2, Table S1). On the other hand, DMSO has a higher dielectric constant (Supplementary Table S2) compared to MeOH which is another influential parameter determining the final fiber diameter.

Moreover, smooth fiber surface morphologies were obtained when either a CHCl_3_:MeOH (18:2) mixture or a CHCl_3_:DMSO (18:2) mixture was spun at 12% RH (Fig. 3b). However once the RH% was increased, the fibers electrospun from CHCl_3_:MeOH (18:2) mixture started to have surface limited pits whereas the ones electrospun from CHCl_3_:DMSO (18:2) mixture were obtained with irregular and more inter-connected surface topographies (Fig. 3b). This can also be seen from the cross-sections of the fibers (Fig. 3b).

Simulations made with the assumptions of the diffusive solvent evaporation from both PCL solutions showed that it took less time for CHCl_3_ to fully evaporate from CHCl_3_:MeOH (18:2) mixture (26.7 a.u.) than from CHCl_3_:DMSO (18:2) mixture (33.3 a.u.) (Fig. 3c,d). This led to less time for thermodynamic instabilities and for fiber drawing to take place. Additionally, MeOH with a higher value of vapor pressure (Table S1) evaporated faster than DMSO.

Latent heat loss accompanying solvent evaporation drives a temperature decrease on the polymer jet surface. The volatility of the solvents defines the extent of this decrease. The simulations showed that the initial *T*_*wb*_ of the CHCl_3_:MeOH (18:2) mixture was lower (−4.1 °C) compared to the CHCl_3_:DMSO (18:2) mixture (−3.5 °C), which was in agreement with the fact that the higher the volatility of a solvent, the lower the resulting *T*_*wb*_. The solvent evaporation resulted in increasing *T*_*wb*_ of CHCl_3_:DMSO (18:2) while only a slight increase was obtained for CHCl_3_:MeOH (18:2) mixture. Additionally, since the *T*_*dp*_ of air at 12% RH is −10.3 °C, Δ*T*(*T*_*dp*_ − *T*_*wb*_) of both solutions were below zero from the beginning. On the other hand, *T*_*wb*_ of CHCl_3_:MeOH (18:2) mixture was below the *T*_*dp*_ both at 35% RH (4.1 °C) and 55% RH (10.7 °C) which yielded positive values of Δ*T* and thus water condensation can be expected. In the case of CHCl_3_:DMSO (18:2) mixture however, *T*_*wb*_ was rising above the *T*_*dp*_ both at 35% and 55% RH conditions after 26.7 (a.u.) (Fig. 3h), which refers to the absence of water condensation on the jet after that point.

Solvent evaporation from a solution prepared in a mixture of solvent and non-solvent alters the solubility parameters of the mixture and thus, its compatibility with the polymer changes. This change can be examined in terms of RED change. The simulations revealed that RED value of CHCl_3_:MeOH (18:2) mixture was never above 1 which was defined as the solubility limit of a polymer in a given solvent. Therefore a RED value exceeding 1 causes a subsequent phase separation of the polymer solutions, which is composed of solvent:non-solvent mixtures, into polymer rich and polymer lean phases. On the other hand, CHCl_3_:DMSO (18:2) mixture RED value started at a higher value than the one of CHCl_3_:MeOH (18:2) and it raised up over 1.

Furthermore, in order to investigate and compare the crystal structure and the crystallinity of these electrospun fibers, WAXS analyses were performed. The WAXS data confirmed the expected semi-crystalline structure previously reported for PCL^48^, as evidenced by the scattering corresponding to the crystalline orthorhombic phase^49, 50^ and by the additional broad peak corresponding to the amorphous phase. The polymer crystallinity was found to be similar for all of the investigated samples revealing no significant influence of the used solvent systems and the applied RH and the respective surface structuring processes with respect to the ratio of amorphous to crystalline phases (Supplementary Fig. S3).

### Influence of solvent to non-solvent ratio and RH on fiber morphologies

**Figure 4:**
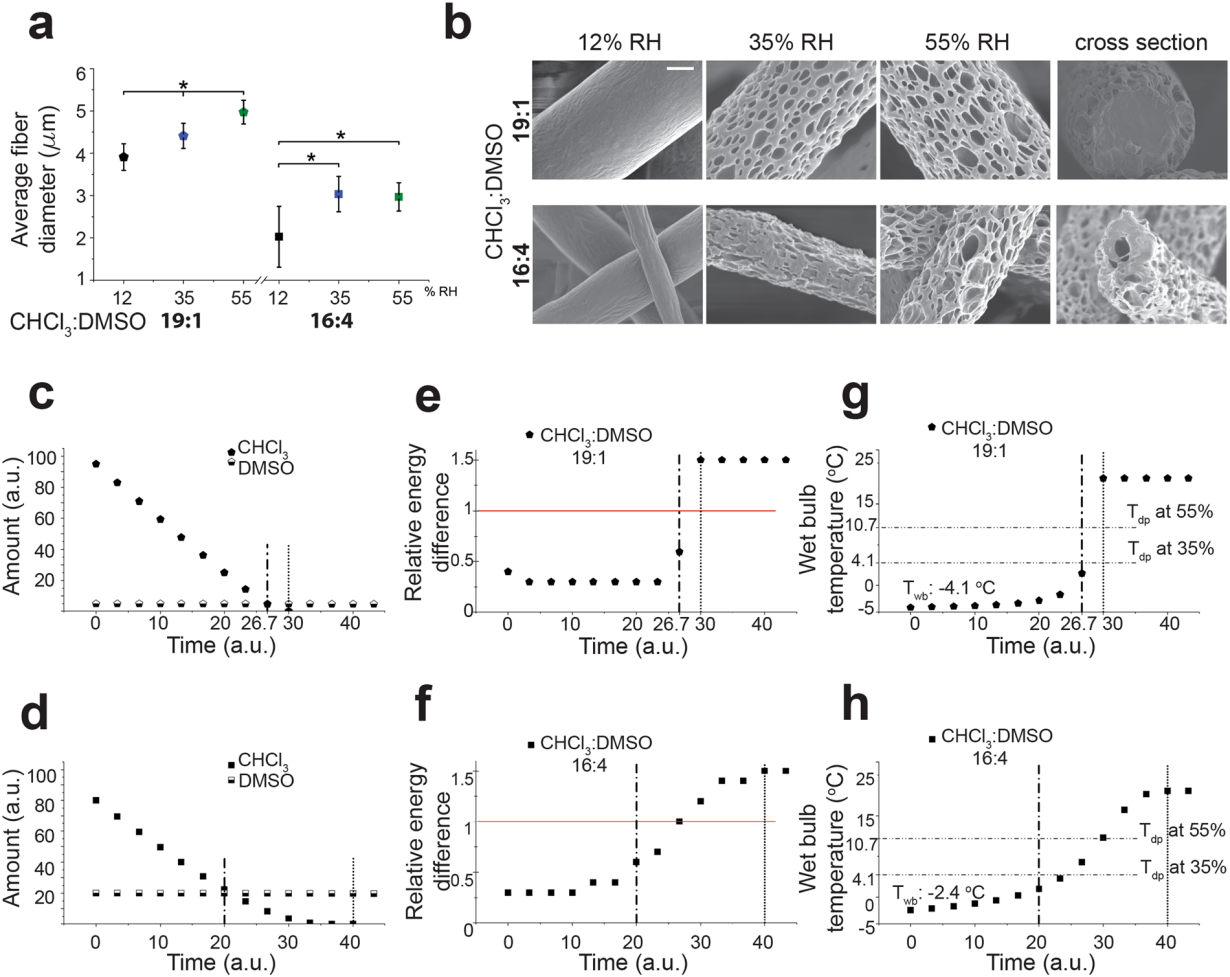
Resulting PCL fiber morphologies, simulated solvent evaporation and RED value changes during fiber formation for the solvent systems CHCl_3_:DMSO: (**a**) Average fiber diameter of electrospun fibers from CHCl_3_: DMSO with 19:1 and 16:4 ratios, Data represent means ± s.d. *P < 0.05; ANOVA One-way test, (**b**) SEM micrographs of electrospun fibers from CHCl_3_: DMSO solutions with 19:1 ratio (upper row) and 16:4 ratio (lower row) at 12, 35 and 55% RH, respectively (scale bar: 1 µm), (**c**,**d**) simulated evaporation of CHCl_3_ and DMSO from PCL solutions with 19:1 and 16:4 ratio, respectively (assuming the initial total amount of the solution is 100 parts and the solvent amount changes by evaporation during electrospinning), (**e**,**f**) simulated RED value change during solvent evaporation from CHCl_3_: DMSO solutions with 19:1 and 16:4 ratios, respectively, (**g**,**h**) wet bulb temperature change (°C) during solvent evaporation from CHCl_3_:DMSO solutions with 19:1 and 16:4 ratio, respectively, (dash-dot line) last time point of similar amounts of solvent (S) and non-solvent; (round dot line) time point S = 0; (red line) solubility limit for the polymer in the solvent system).

In order to examine the final fiber morphology change in relation to non-solvent amount in the solvent mixture, we electrospun PCL solutions prepared in CHCl_3_:DMSO (19:1) (with initial RED of 0.4) and CHCl_3_:DMSO (16:4) (with initial RED of 0.3) mixtures again at three different RH conditions. Varying the DMSO amount in the solution mixture altered the average fiber diameter (Fig. 4a) as well as surface and inner morphology (Fig. 4b). The increase of the amount of DMSO in the CHCl_3_:DMSO mixture from 19:1 to 16:4 resulted in significantly lower average fiber diameter at each RH condition. One can further elaborate with various ratios of solvent and non-solvent by keeping in mind that there is an upper limit of the amount of non-solvent and hence to the factor that is decreasing the fiber diameter. Moreover, with increase of RH, an increase of fiber diameter was observed for same solution compositions (Fig. 4a).

The fiber surface topographies were evolving in a similar manner in dependency of RH as found for the PCL solutions prepared in CHCl_3_:MeOH (18:2) and CHCl_3_:DMSO (18:2) mixtures. At low RH, both PCL solutions prepared in CHCl_3_:DMSO (19:1) and CHCl_3_:DMSO (16:4) mixtures had smooth surfaces whereas fibers with structured surfaces were obtained at higher RHs (Fig. 4b).

The simulations of the solvent evaporation showed that complete loss of chloroform was achieved by 30 time units (a.u.) and it was delayed by the increase of DMSO amount in the solution to 33.3 time units (a.u.) for 18:2 mixture and to 40 time units (a.u.) for 16:4 mixture (Figs 3d and 4c,d).

Furthermore, the initial *T*_*wb*_ of the solutions increased in value with increasing amounts of DMSO in the solution from −4.1 °C to −3.5 °C and finally to −2.4 °C (Figs 3h and 4g,h) as expected due to the volatility differences of the mixtures.

The solubility change of PCL by the evaporation of solvents was investigated in relation to the change in the RED value (Fig. 3f and 4e,f). RED is reaching the value of 1 and crossing this solubility limit at an earlier time point with increasing amounts of DMSO, which indicates an earlier phase separation of the solutions.

### Solvent- polymer interaction during fiber formation and its relation to fiber surface morphology for PLLA and PVP

**Figure 5:**
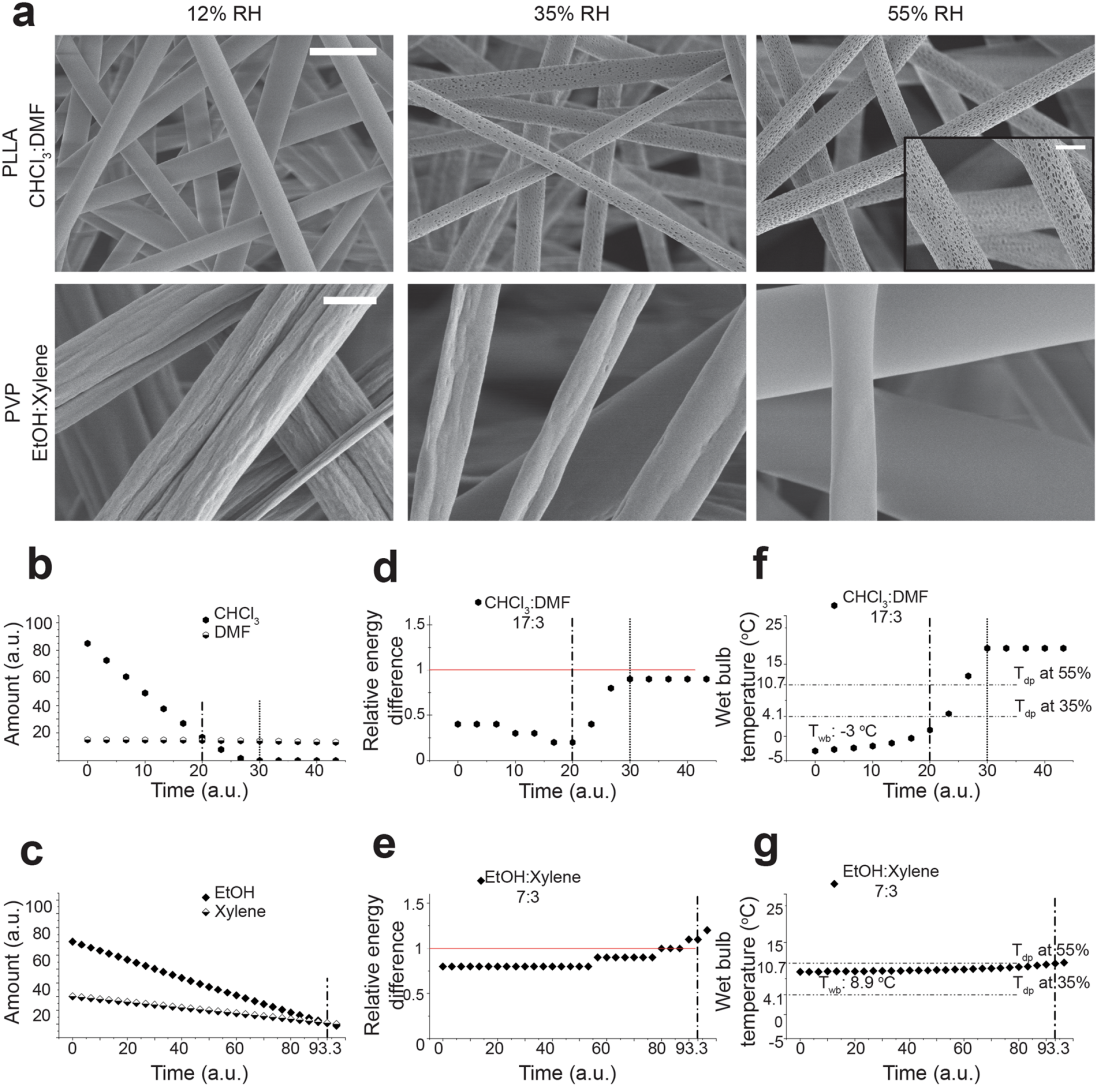
Resulting PLLA and PVP fiber morphologies, Simulated solvent evaporation and RED value changes during PLLA and PVP fiber formation: (**a**) SEM micrographs of PLLA electrospun fibers from CHCl_3_:DMF (17:3) solution (upper row) and PVP electrospun fibers from EtOH:Xylene (14:6) solution (lower row) at 12, 35, 55% RH, respectively (scale bar: upper row 5 µm, inlet 2 µm, lower row 1 µm), simulated evaporation of (**b**) CHCl_3_ and DMF and (**c**) EtOH and Xylene from PLLA and PVP solutions, respectively (assuming the initial total amount of a solution is 100 parts and the solvent amount changes by evaporation during electrospinning), simulated RED value change during solvent evaporation from (**d**) CHCl_3_:DMF and (**e**) EtOH:Xylene solutions, wet bulb temperature change (°C) during solvent evaporation from (**f**) CHCl_3_:DMF and (**g**) EtOH:Xylene solutions, (dash-dot line) last time point of similar amounts of solvent (S) and non-solvent; (round dot line) time point S = 0; (red line) solubility limit for the polymer in the solvent system.

In order to investigate the universality of our approach, we have also demonstrated how electrospun PLLA (Fig. 5a, Supplementary Fig. S4a) as well as PVP (Fig. 5a, Supplementary Fig. S5a) fiber surface structure formation correlates with theoretically predicted polymer solubility alterations in different solvents during their evaporation, the corresponding solvent evaporation rates and subsequent polymer jet surface temperature changes (Fig. 5b‒g, Supplementary Figs S4 and S5). The pore morphology on both PLLA and PVP electrospun fibers was influenced collectively by the RED and *T*_*wb*_ change during solvent evaporation as was observed for PCL. Surface topography evolution was found to be similar for PLLA and PCL, while the opposite trend was observed for PVP as expected due to the polymer’s hydrophilic nature. These findings suggest that, for all three polymers, the same conclusions can be drawn with respect to influencing factors such as environmental humidity, non-solvent volatility in comparison to the solvent and solvent to non-solvent ratio. Further discussion and results can be found in Supplementary Information.

### Fibrous membranes as oxygen-sensing scaffolds

To highlight the advantage of the fibrous morphology of electrospun PCL membranes for optical sensing applications, we have investigated their suitability for physical entrapment of an indicator dye and their subsequent use as oxygen sensor in 3-D cell cultures. We have chosen a highly hydrophobic luminescent dye (PtTFPP) due to its inherent high photostability, excellent brightness (defined as the product of phosphorescent or fluorescent quantum yield and the molar extinction coefficient), as well as the possibility of oxygen calibration by luminescence lifetime reading in the microsecond range^51, 52^. Lifetime readout is generally preferred to intensity-based calibration since the measurements are independent of dye concentration and it allows a higher robustness and reproducibility of the oxygen sensing performance^52^.

The physical entrapment of PtTFPP has been obtained by choosing a mixture of acetone and water as non-solvents for PCL at room temperature with the former being a ‘good’ solvent and the latter being a non-solvent for the luminescent dye^44^. Upon evaporation, the solution is enriched in water, thus the dye diffuses through the membrane and precipitates within the mesh structure.

**Figure 6:**
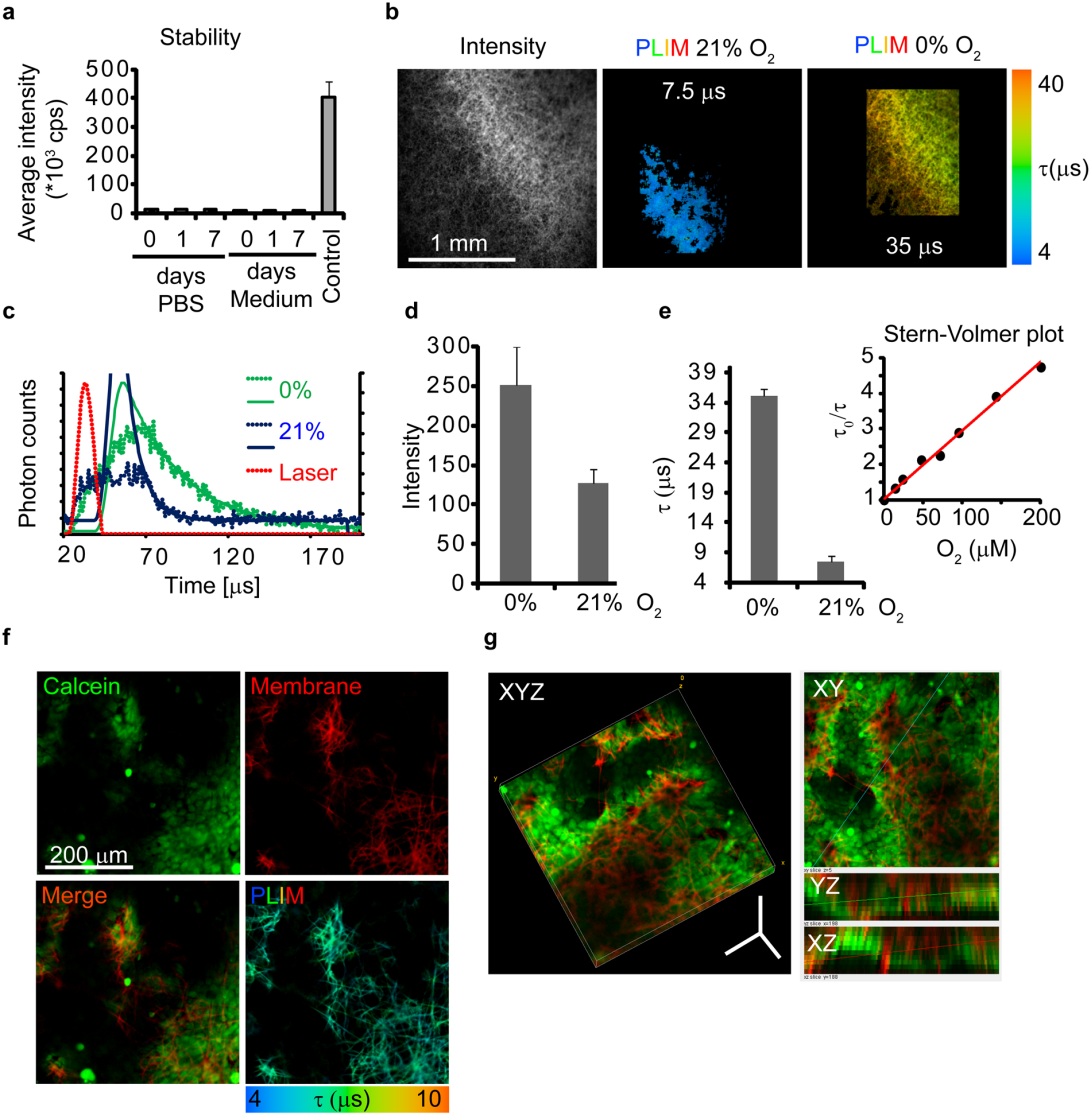
Application of fibrous electrospun membranes to O_2_ sensing: (**a**) Stability of impregnated PtTFPP in PBS and cell culture medium during incubation at 37 °C. (**b**) Intensity map and phosphorescence lifetime imaging of stained membrane in PBS buffer at 21 kPa O_2_ and after deoxygenation obtained by confocal PLIM. (**c**) Representative phosphorescence decay curves at 0 and 21% O_2_. (**d**,**e**) average phosphorescence intensities and lifetimes measured at 0 and 21% O_2_, respectively and Stern-Volmer relationship. (**f**) Confocal PLIM of live HCT116 cells cultivated on membranes. Images of cells (stained with Calcein Green) and membrane (red) and in PLIM (membrane only) are shown. (**g**) 3-D reconstruction of cells growing in membrane. Different projections are shown. Scale bar is in µm.

The PtTFPP-stained membranes, which were made of porous microfibers, were found to be bright and stable. When incubated in PBS and cell growth medium for up to 7 days at 37 °C, we did not observe leakage of the dye and detected only minor phosphorescence readings of 10–12 × 10^3^ cps in solution, compared to >400 × 10^3^ cps for the membrane (Fig. 6a).

The high brightness of the stained membranes was confirmed by analysis on confocal microscope, where we observed strong signals upon excitation at the minor Q band of PtTFPP at 540 nm (emission collected at 650 nm). Compared to ‘blue light’ excitation (Soret band), 540 nm excitation mode is less destructive for cells and is more convenient for filtering out the biological autofluorescence^44^. Analysis of membranes at different O_2_-concentrations confirmed that they are suitable for both in phosphorescence intensity and lifetime readouts (Fig. 6b–e).

It was recently demonstrated that a hybrid O_2_ sensing scaffold developed by use of commercial polystyrene-based Alvetex^TM^ membranes stained with PtTFPP had phosphorescence lifetime of 56.5 μs at 0 kPa O_2_ and 20 μs at 21 kPa O_2_^44^. PCL nanoporous membranes instead showed higher quenching effect with approximately five-fold change of phosphorescence lifetime.

In addition, linear Stern-Volmer relationship was observed for PCL membranes (Fig. 6)^53^. This is a unique finding for this type of systems and it is probably related to the higher accessibility of the dye to the quenching analyte due to the highly porous matrix morphology. This feature can be translated to steady sensitivity and accuracy of oxygen monitoring of cell grown in the 3-D structure.

For comparison, we also stained smooth micro- and nanofiber PCL membranes with PtTFPP dye and evaluated them in solution. All tested types of membranes were sufficiently bright and photostable in PLIM microscopy, displaying comparable O_2_-sensitivity (Supplementary Fig. S6). However, membranes with porous microfibers were the only ones demonstrating linear (R^2^ = 0.9903) Stern-Volmer relationship.

Finally, the compatibility of the membranes impregnated with PtTFPP for biological experiments was investigated. Human colon carcinoma cells HCT116 were seeded and cultivated on membranes for 48 hours, stained with Calcein Green viability dye and live cells were imaged using confocal PLIM (Fig. 6f,g). Cells adhered to all types of membranes and remained viable during the experiment (48 hours; Supplementary Fig. S7). In addition, cells accumulated not only at the surface of the membrane but also within, growing into the 3-D environment. Using PLIM, we observed that phosphorescence lifetime values at the surface and 30–50 µm deep at 21 kPa O_2_ were stabilized to values around 7–8 µs, indicating that membranes provided efficient O_2_ diffusion due to the highly porous fiber structure.

Thus, PCL membranes with different surface morphologies can be used for successful impregnation of the hydrophobic O_2_-sensitive indicator dye, PtTFPP. They additionally show cytocompatibility, allowing the investigation for growth and analysis of oxygenation of 3-D cell cultures. For O_2_-sensing applications, we found microfibers with porous surface morphology being the most suitable (linear Stern-Volmer calibration), compared with nanofiber and smooth microfiber materials. Hence, these electrospun fibrous membranes represent an interesting alternative to hybrid PS Alvetex^TM^ and related 3-D scaffolds as their production is inexpensive, easy and up-scalable^54^.

## Discussion

Electrospun fibers with tailored surface topographies can be generated by adapting solution parameters such as solvent system and polymer molecular weight and/or environmental parameters such as temperature and relative humidity^8, 13, 18–20, 27, 29, 55^. Hence, it is a prerequisite to select appropriate solvents to obtain the desired surface morphology at a defined environmental condition. To reach a more universal theoretical approach for the complex electrospinning process, we utilized HSP to determine not only the initial polymer-solvent compatibility but more importantly the solubility changes for the model polymer PCL during solvent evaporation throughout the electrospinning process. The findings were then also tested for PLLA and PVP to gain deeper insights on the applicability of the tool for other polymers.

During electrospinning, a simultaneous mass transfer of evaporating solvents within the jet and the penetration of water from the environment takes place and the resulting fiber morphology is determined by the competition between the rapid phase separation and solidification^56^. To date, the hypotheses of evaporative cooling and phase separation yielding surface topographies on electrospun fibers have never been correlated in a theory-driven approach to understand, predict and control the surface texturing of e-spun fibers in relation with *T*_*dp*_ of the electrospinning environment and simulated *T*_*wb*_ on the polymer jet surface. By investigating the electrospun fibers produced from solutions of PCL in two different good solvents (CHCl_3_ and DCM) and respective calculation of the *T*_*wb*_’s, it was concluded that the higher the solvent volatility, the lower the *T*_*wb*_ (Fig. 2). Additionally, raising the environmental RH increased the *T*_*dp*_. In turn, the difference between these two temperatures determined the degree of water condensation on fiber surfaces and the further texturing of the fiber surface.

Moreover, solvent and non-solvent mixtures were electrospun at varied RH conditions to explore the resulting fiber morphology and the thermodynamic instability-driven phase separation of polymer solutions. Decreasing temperature due to loss of solvent and simultaneous change in RED value determined the degree of phase separation which in turn affected the apparent irregularity and depth of surface structures. The smooth fiber surface morphology obtained from CHCl_3_:MeOH (18:2) mixture at 12% RH can be explained with the negative value of Δ*T* corresponding to lack of water condensation as well as a RED value below 1 (Fig. 3e and g). Besides, the surface limited pits on the fibers electrospun from the same mixture at 35% and 55% RH are a collective result of the solvent evaporation rate differences and the consequent change in the *T*_*wb*_ and RED (Fig. 3c,e and g). On the one hand, positive Δ*T* leading to water condensation triggers phase separation of the polymer solution as a non-solvent. On the other hand, the amount of water soluble non-solvent available in the jet is decreasing by time (Fig. 3c) and there is less time available till it solidifies to generate the non-solvent induced phase separation. Additionally RED value is not crossing over 1 before it solidifies.

CHCl_3_:DMSO (18:2) mixture at 12% RH had also negative Δ*T* referring to no water condensation and thus smooth surface morphologies were obtained. However, its RED value increased to higher values than that of CHCl_3_:MeOH (18:2) mixture, although this increase was not high enough to induce phase separation at low RH condition by itself. The irregular and inter-connected surface pores obtained at 35% and 55% RH can be the synergistic result of positive Δ*T* values till increasing *T*_*wb*_ reached to *T*_*dp*_ with an increasing RED value in the meantime (Fig. 3f and h). As a hygroscopic non-volatile solvent, DMSO might have enabled more water penetration into the jet at these higher RH conditions and thus the solution might have already reached even a higher RED value than the simulated one.

Additionally, the comparison of DMSO amounts in the solution mixture revealed that at the highest RH condition, a more pronounced structuring of the fibers was obtained due to an increase of phase separation deriving from higher DMSO concentrations (Figs 3b and 4b). The higher dielectric constant and low volatility of DMSO increased the drawing ratio of the ejected jet, which in turn decreased the final fiber diameter. The increased amount of humidity, however acted in the opposite way by possibly accelerating a phase separated skin formation limiting the drawing process of the jet such as found by Medeiros *et al*. for PS and PMMA in THF, DMF and toluene^5555^ thus the fiber diameter increased with increased RH. Certainly, a loss of surface texturing might be considered as soon as the drawing process is dominating the fiber formation process. An additional drawing process, e.g. caused by up-take of the fiber on a fast rotating drum, is decreasing the fiber diameter with a partial loss of surface structures. We observed this with aligned PCL fibers electrospun onto a rotating drum from the same solution that yields porous fibers when collected on a static plate collector (data not shown).

**Figure 7:**
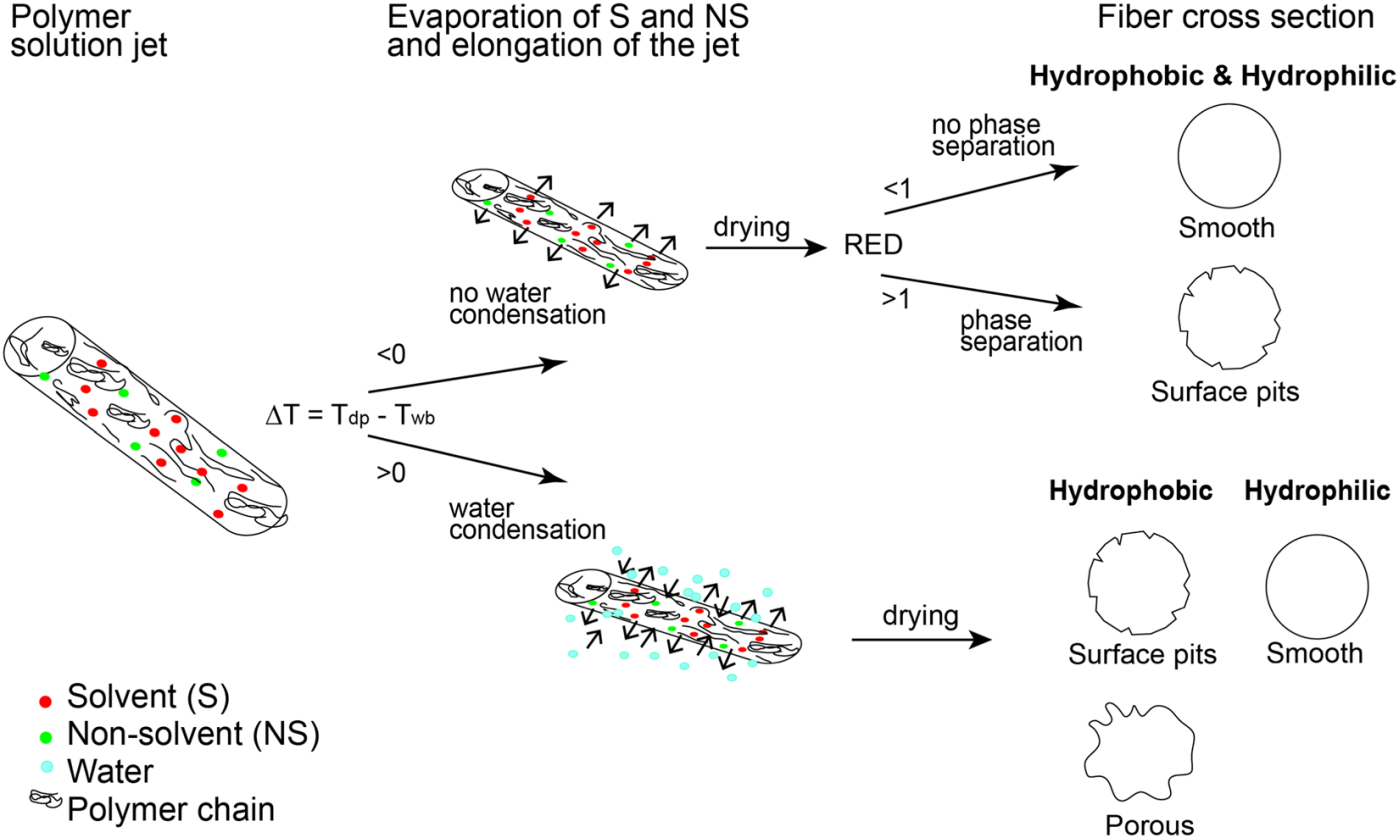
Depiction of pore formation mechanism for the electrospinning process.

Overall, we propose the pore formation mechanism as depicted in Fig. 7.

## Conclusion

In summary, the surface structure formation on electrospun PCL fibers originates from the interplay between polymer solubility changes and jet surface temperature decrease deriving from differences in solvent volatility. The synergistic effect of these parameters together with the relative humidity defines the extent of surface structuring on electrospun PCL fibers as well as PLLA and PVP fibers.

These findings hold great potential to extend the tool-box for tailoring the topographies of electrospun fibers with better efficiency, control and reproducibility for surface sensitive applications such as drug delivery, advanced tissue engineering, catalysis, filtration and biosensing technology as demonstrated with O_2_ sensing in 3-D cell culture in this study.

## Electronic supplementary material

Supplementary PDF File
