# Supplementary material for: Steering surface topographies of electrospun fibers: understanding the mechanisms
Source: Sci Rep. 2017 Mar 13;7:158. doi: 10.1038/s41598-017-00181-0 (PMC5427888; doi:10.1038/s41598-017-00181-0)
Supplement: Supplementary file 1 — Supplementary PDF File [file 41598_2017_181_MOESM1_ESM.pdf]

Supplementary Information

Steering surface topographies of electrospun fibers: understanding the mechanisms

*Gökçe Yazgan, Ruslan I. Dmitriev, Vasundhara Tyagi, James Jenkins, Gelu-Marius Rotaru, Markus Rottmar, René M. Rossi, Claudio Toncelli, Dmitri B. Papkovsky, Katharina Maniura-Weber, Giuseppino Fortunato**

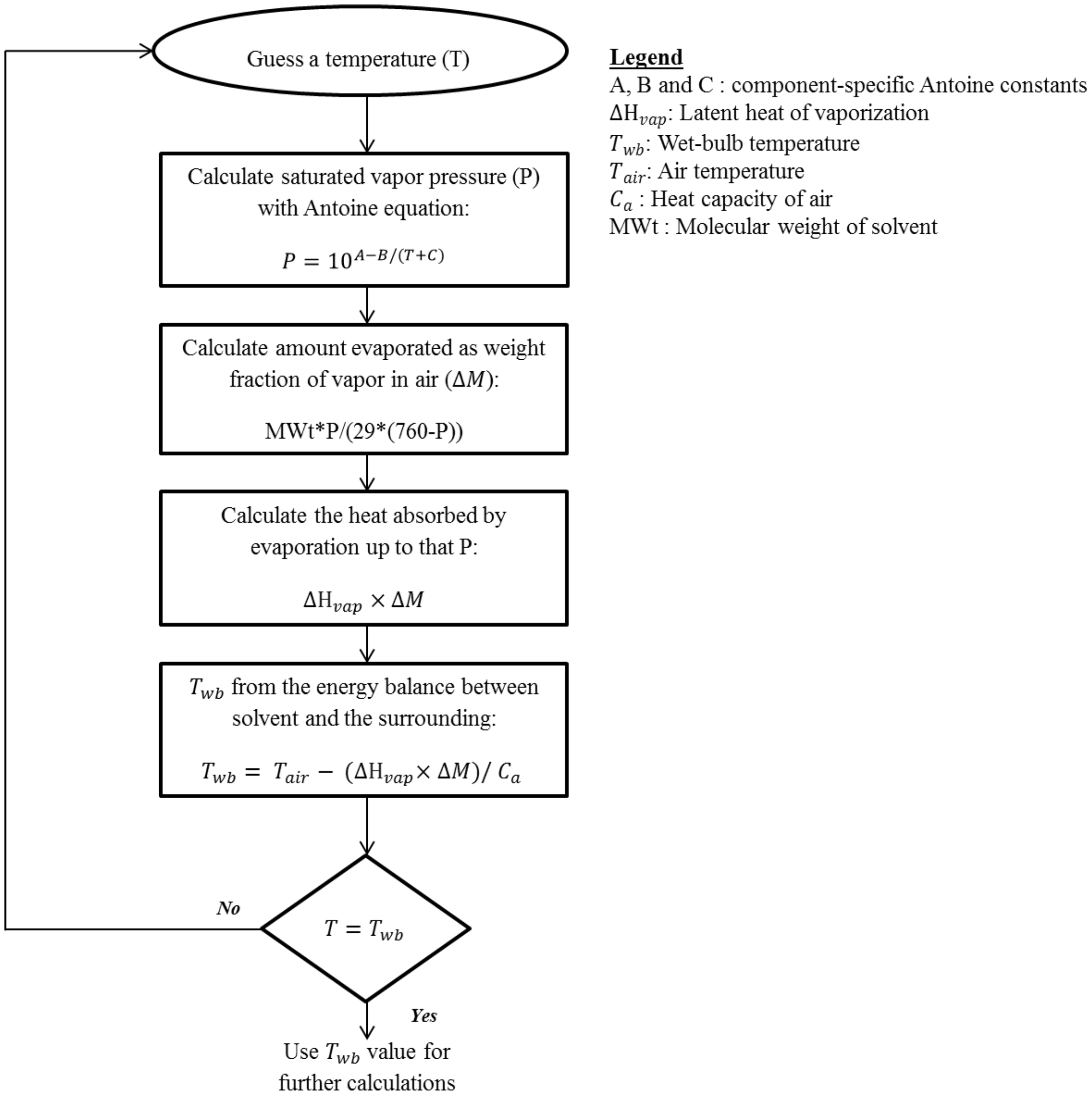

Figure S1. Iterative wet bulb temperature calculation

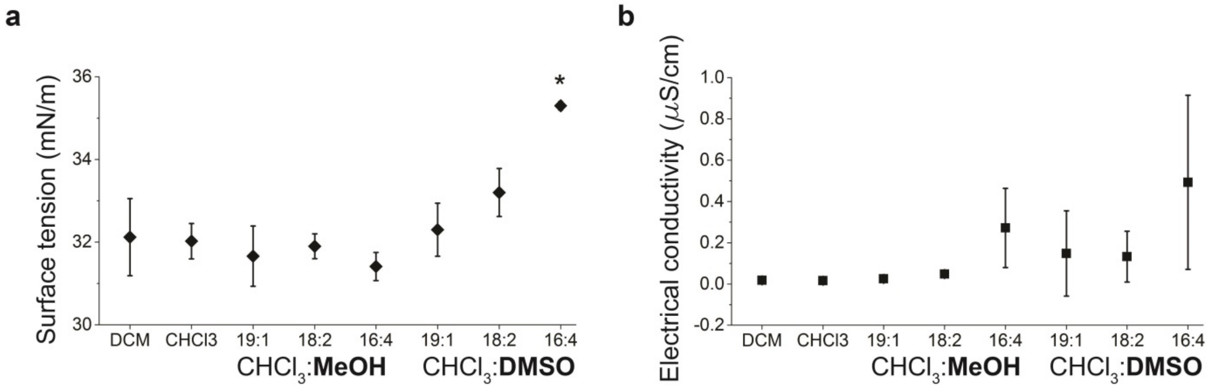

Figure S2. PCL solution properties: a) surface tension and b) electrical conductivity of PCL solutions prepared in DCM, $CHCl_3$, mixture of $CHCl_3$:MeOH with ratios of 19:1, 18:2 and 16:4 and mixture of $CHCl_3$:DMSO with ratios of 19:1, 18:2 and 16:4. Data represent means ± s.d. * P<0.05; ANOVA One-way test.

Table S1. Rotational rheological measurement (measured at 20°C) of spinning solutions

	Shear rate		
Solvents	0.01 s^{-1}	0.1 s^{-1}	1 s^{-1}
CH_2Cl_2	2.5 Pa.s	4.3 Pa.s	5.5 Pa.s
$CHCl_3$	1.4 Pa.s	1.8 Pa.s	2.3 Pa.s
$CHCl_3$:DMSO 9:1	1.2 Pa.s	1.4 Pa.s	1.5 Pa.s
$CHCl_3$:MeOH 9:1	1.7 Pa.s	2.2 Pa.s	2.8 Pa.s

Table S2. List of solvents used for HSP solubility verification and their selected physical properties

Solvent	Boiling Point [°C] at 750 mmHg	Vapor Pressure [mmHg] at 21°C	Solubility in water [w/w %] at 25°C	Dielectric constant	Electrical conductivity [S/cm]	Surface Tension [dyn/cm] at 20°C
DCM	40	376	1.3	9.1	4.3E-11	28.1
$CHCl_3$	61	169	0.8	4.8	1.0E-10	27.2
DMSO	189	0.7	total	46.6	2.0E-09	43.7
EtOH	78	46	total	22.4	1.4E-09	22.3
Acetonitrile	82	71	total	37.5	6.0E-10	29.1
MeOH	64	103	total	32.6	1.5E-09	22.6

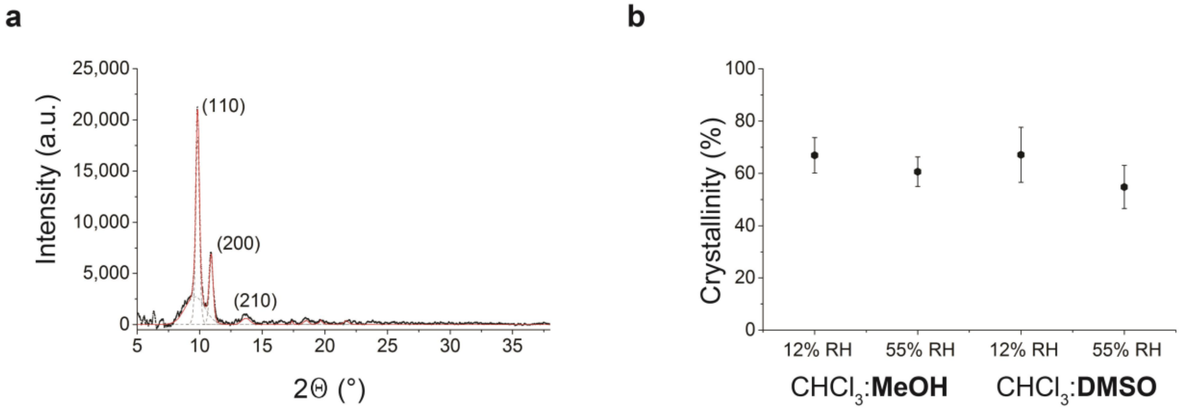

Figure S3. Wide angle X-ray scattering (WAXS) results: a) Typical WAXS pattern of PCL electrospun fibers. Full circles show experimental data points, dashed lines represent Pearson7 peak fits and solid line is the sum of the fit. The indexed peaks correspond to the crystalline orthorhombic phase of PCL. b) WAXS crystallinity calculated by taking the ratio of crystalline diffraction area to the total area for PCL fibers electrospun at 12 and 55% RH from $CHCl_3$: MeOH and $CHCl_3$:DMSO solutions.

Solvent- polymer interaction during fiber formation and its relation to fiber surface morphology for PLLA and PVP. To explore the universality of HSP based calculations of solvent-polymer interactions during electrospinning and the correlation with the resulting electrospun fiber surface morphologies, two additional polymers were studied. First PLLA solutions prepared with solvent mixtures of $CHCl_3$:MeOH (18:2 and 17:3) and $CHCl_3$:DMF (17:3) were compared in addition to PCL fibers. The initial RED values of the solvent mixtures with respect to PLLA were calculated as 0.5 for both $CHCl_3$:MeOH solutions and as 0.4 for $CHCl_3$:DMF solution. The values <1 indicated that the polymer is initially soluble in all three solvent systems. All solutions were electrospun at 12, 35 and 55% RH conditions and the surface morphologies of the resulting fibers were compared with the simulated solvent evaporation, RED value as well as jet surface temperature changes during evaporation.

The fiber surface topographies were evolving in dependency of RH in the same manner as was found for the PCL solutions. At low RH, PLLA solutions prepared in $CHCl_3$:DMF (17:3) mixture yielded smooth surfaces whereas fibers with slightly structured surfaces were obtained from PLLA solution prepared in $CHCl_3$:MeOH (18:2) and $CHCl_3$:MeOH (17:3). All fibers had an increase of surface structures at higher RHs (Figure S4a). The type of surface structures also differed from $CHCl_3$:MeOH to $CHCl_3$:DMF spun fibers similar to what could be observed for PCL. $CHCl_3$:MeOH solutions yielded surface pits while $CHCl_3$:DMF solutions yielded surface holes. These structures are again well correlated with the simulated values of solvent evaporation and the resulting RED as well as T_{wb} change. For instance, although the initial T_{wb} of -3.7°C is above the dew point of -10.3°C at 12% RH (Figure S4g), which means that no water condensation was expected, the fibers had slight surface structures. However, the RED value for $CHCl_3$:MeOH (17:3) goes beyond the solubility limit of 1 and reaches 1.7 during evaporation (Figure S4e), which presumably is the reason for phase separation and for the subsequent pore formation. Moreover, $\Delta T = (T_{dp} - T_{wb})$ of all solutions was above zero for the increased RH% conditions which in turn yielded structured fibers.

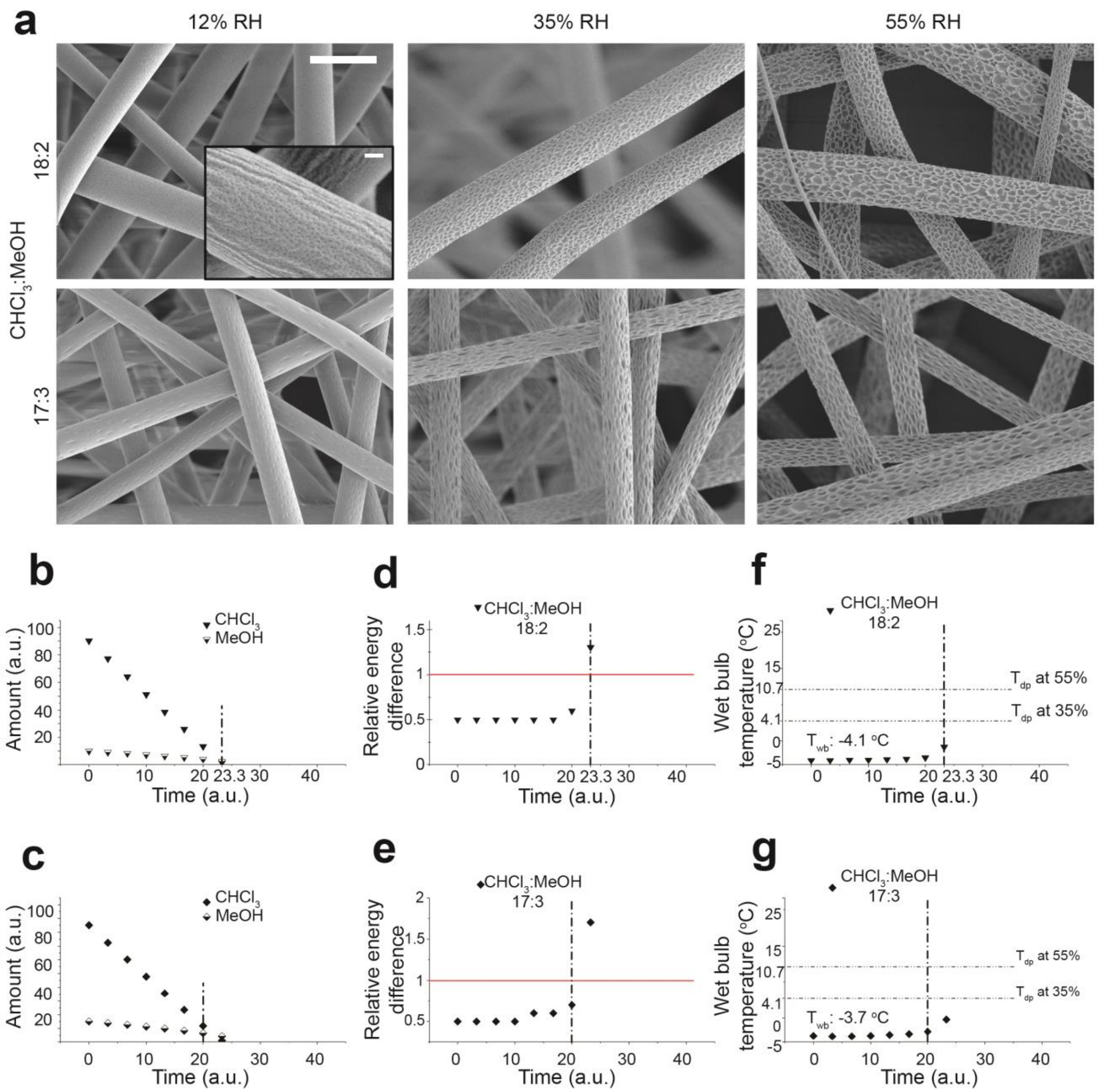

Figure S4. Resulting PLLA fiber morphologies, Simulated solvent evaporation and RED value changes during PLLA fiber formation for the solvent systems of $CHCl_3$:MeOH: SEM micrographs of electrospun fibers from a) $CHCl_3$:MeOH (18:2) and $CHCl_3$:MeOH (17:3) solutions at 12, 35, 55 % RH, respectively (scale bar: 5μm, insert 0.5 μm), simulated evaporation of $CHCl_3$ and MeOH from PLLA solutions with solvent ratios of b) (18:2) and c) (17:3), respectively (assuming the initial total amount of a solution is 100 parts and the solvent amount changes by evaporation during electrospinning), simulated RED value change during solvent evaporation from

$CHCl_3$:MeOH d) (18:2) and e) (17:3) solutions, respectively, wet bulb temperature change (°C) during solvent evaporation from $CHCl_3$:MeOH f) (18:2) and g) (17:3) solutions, respectively, (dash-dot line) last time point of similar amounts of solvent (S) and non-solvent; (round dot line) time point S=0; (red line) solubility limit for the polymer in the solvent system.

Secondly, PVP solutions prepared with EtOH alone (Supplementary Fig. S5) and with a solvent mixture containing a non-solvent (EtOH:Xylene (7:3)) were investigated (Figure 5). The initial RED values with respect to PVP were calculated as 0.6 for EtOH solution and as 0.8 for EtOH:Xylene solution, which confirmed the polymer's solubility in the respective solvents. Both solutions were electrospun at 12, 35 and 55% RH conditions and their corresponding surface morphologies were compared with the simulated solvent evaporation, RED value as well as jet surface temperature changes during evaporation. The fibers electrospun from pure EtOH were smooth in all three RH conditions with an increase in the homogeneity and circularity with RH (Supplementary Fig. S5a). On the other hand, at low RH conditions, EtOH:Xylene solution yielded fibers with indentations on their surface apart from the wrinkled morphology and with smooth surface in a more circular morphology at higher RH (Figure 5a). These structures were well correlated with the simulated values of solvent evaporation and the consequent RED as well as T_{wb} change. For instance, EtOH as a good solvent resulted in a constant RED value <1 (Supplementary Fig. S5c) during solvent evaporation hence no contribution to any phase separation could occur. Besides, as the T_{wb} value (6.9°C) was constantly below the dew point of 10.7°C at 55% RH (Supplementary Fig. S5d), the resulting condensed water acted as a secondary solvent and more homogeneous and circular fibers were obtained. In the case of fibers electrospun at 55% RH from non-solvent (xylene) containing solution, unlike the hydrophobic PCL and PLLA fibers, hydrophilic PVP fibers had smooth fiber surfaces due to the condensed water being a good solvent for the polymer (Figure 5a). On the other hand, structured surfaces obtained at lower RH were a consequence of RED values exceeding the solubility limit of 1 (Figure 5e) when electrospun from xylene containing PVP solution.

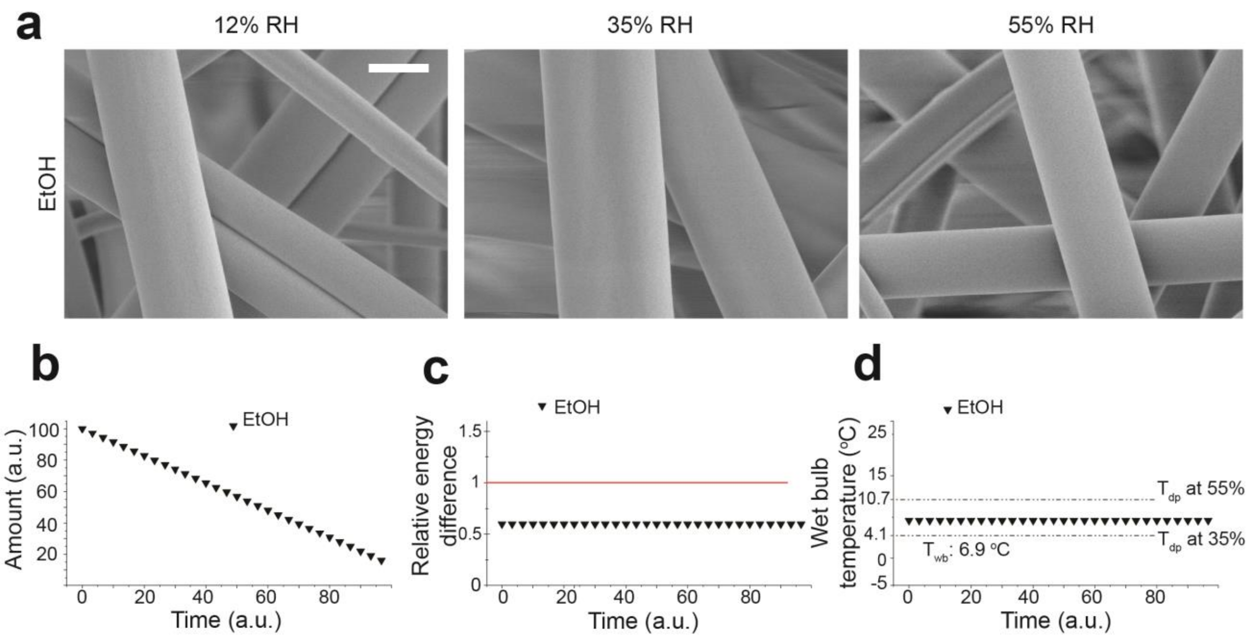

Figure S5. Simulated solvent evaporation and RED value changes during PLLA fiber formation for the solvent systems $CHCl_3$:MeOH and $CHCl_3$:DMF: a,b,c) simulated evaporation of $CHCl_3$ and MeOH and $CHCl_3$ and DMF, respectively from PLLA solutions (assuming the initial total amount of a solution is 100 parts and the solvent amount changes by evaporation during electrospinning), d,e,f) simulated RED value change during solvent evaporation from $CHCl_3$:MeOH and $CHCl_3$:DMF solutions, respectively g,h,i) wet bulb temperature change (°C) during solvent evaporation from $CHCl_3$:MeOH and $CHCl_3$:DMF solutions, respectively, (dash-dot line) last time point of similar amounts of solvent (S) and non-solvent; (round dot line) time point S=0; (red line) solubility limit for the polymer in the solvent system).

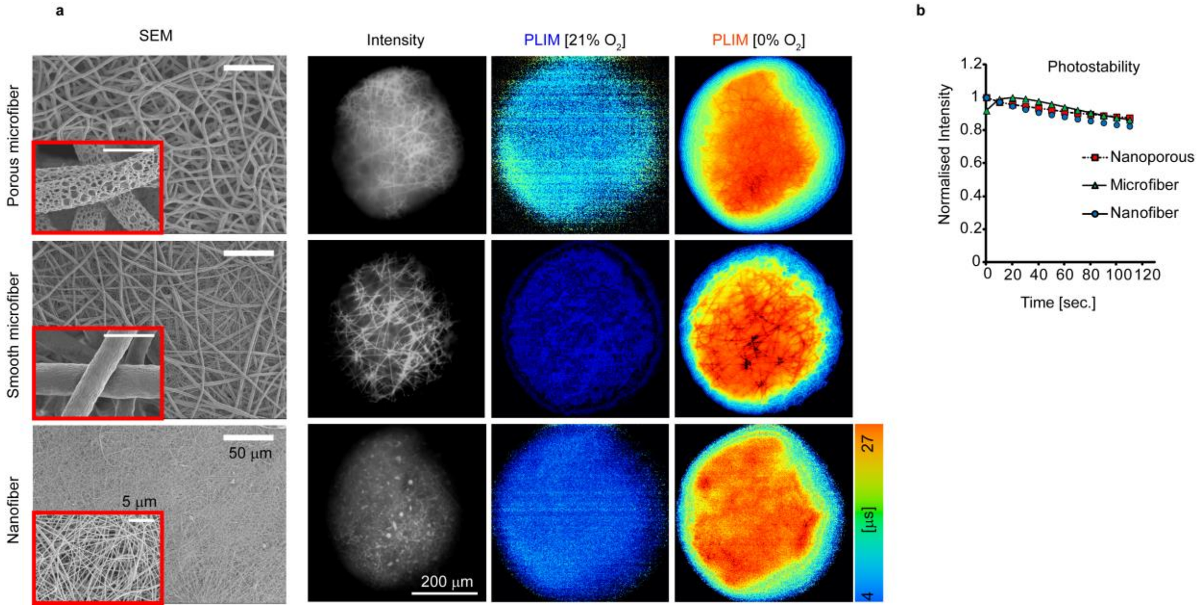

Figure S6. Comparison of O_2-sensing properties between different membrane morphologies. a) SEM micrographs as well as Intensity and phosphorescence lifetime images of stained membranes in PBS buffer at 21 kPa O_2 and after deoxygenation obtained by widefield PLIM. Colormap indicates the range of phosphorescence lifetimes. b) Comparison of photostability of stained membranes upon continuous illumination with 20 mW LED 390 nm (emission collected at 650 nm).

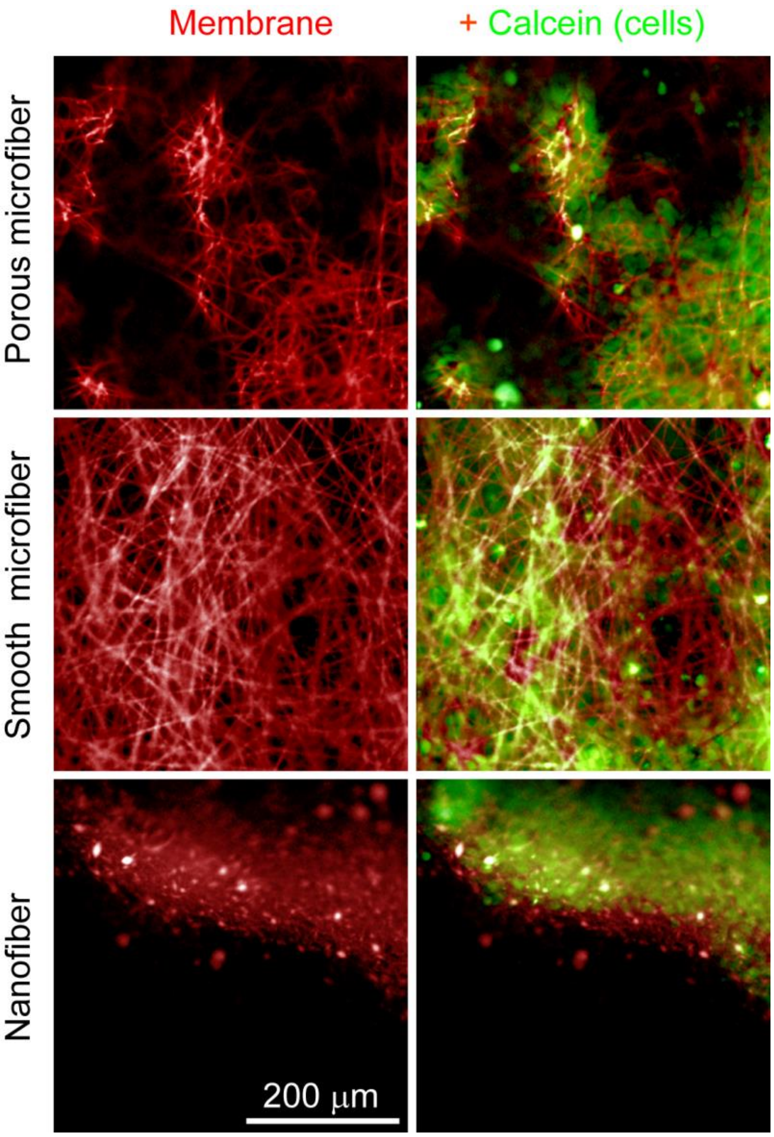

Figure S7. Comparison of cells attachment to the PtTFPP-stained membranes with different surface morphologies. Confocal fluorescence images of live cells (stained with Calcein Green) grown on membranes (red, exc. 540 nm, em. 650 nm) are shown.
